# Improving the forecasting performance of temporal hierarchies

**DOI:** 10.1371/journal.pone.0223422

**Published:** 2019-10-03

**Authors:** Evangelos Spiliotis, Fotios Petropoulos, Vassilios Assimakopoulos

**Affiliations:** 1 Forecasting and Strategy Unit, School of Electrical and Computer Engineering, National Technical University of Athens, Zografou, Greece; 2 School of Management, University of Bath, Bath, United Kingdom; University of Padova, ITALY

## Abstract

Temporal hierarchies have been widely used during the past few years as they are capable to provide more accurate coherent forecasts at different planning horizons. However, they still display some limitations, being mainly subject to the forecasting methods used for generating the base forecasts and the particularities of the examined series. This paper deals with such limitations by considering three different strategies: (i) combining forecasts of multiple methods, (ii) applying bias adjustments and (iii) selectively implementing temporal hierarchies to avoid seasonal shrinkage. The proposed strategies can be applied either separately or simultaneously, being complements to the method considered for reconciling the base forecasts and completely independent from each other. Their effect is evaluated using the monthly series of the M and M3 competitions. The results are very promising, displaying lots of potential for improving the performance of temporal hierarchies, both in terms of accuracy and bias.

## 1 Introduction

Hierarchical forecasting has attracted the attention of the forecasting community the past few years for two reasons. First, forecasting using hierarchies can enhance the forecasting performance. Suitably selecting the optimal level of aggregation or even optimally combining across multiple levels can reduce the forecast error significantly. Second, and possibly more importantly, hierarchical forecasting renders the forecasts coherent across all hierarchical levels. Even if performance improvements were negligible, the property of coherency is very attractive from a managerial point of view, as it allows aligned decision making across the different functions of an organisation. The literature has shown that these benefits apply to both cross-sectional (product or geographical) [1, 2] and temporal (time) hierarchies [3].

Temporal hierarchies, as well as hierarchical reconciliation in general, base their efficiency on the well-known benefits of forecast combination. Forecasts are produced at the various aggregation levels to form the base, non-coherent forecasts. These base forecasts are then combined using optimal weights based on ordinary or weighted least squares estimators. The product is a new set of forecasts that is coherent across all levels. The above describes a combination function that is limited across the various hierarchical levels. It is still not clear, however, if the performance benefits arise because of the forecast combination itself or because of the fact that these forecasts are produced at different hierarchical levels. While different forecasting methods may be used on different aggregation levels, the literature has not considered the combination of multiple forecasting methods for each aggregation level. We attempt to shed some light on the importance of combining across aggregation levels versus combining methods for each aggregation level versus performing a double combination both across methods and aggregation levels.

One important assumption of forecast reconciliation is that the base forecasts produced at the various aggregation levels are unbiased. However, this is seldom the case in practice. Numerous past studies have based their findings on real data sets and have not accounted for the actual bias of the forecasts (for example, see [3–5]). We empirically explore the effects of the bias of the base forecasts on the accuracy and bias of the final reconciled forecasts by appropriately applying empirical bias-adjustment strategies.

A potential drawback of considering temporal hierarchies, is that the reconciled forecasts tend to be much smoother than the original data due to the effect of averaging across multiple aggregation levels. This is particularly the case for seasonal series, as both seasonal and non-seasonal forecasts may be combined to provide the reconciled ones. Although extrapolating just the original series and using the bottom-up method afterwards to construct reconciled forecasts can mitigate this issue and provide reasonable results, it is still unclear when such an alternative should be preferred over temporal hierarchies. Thus, we empirically investigate the effect of seasonal shrinkage by introducing an heuristic rule that identifies cases where the seasonal components of the examined series could be inadequately captured by temporal hierarchies, making appropriate decisions.

In summary, our contribution is threefold:

- We evaluate the performance of combination across methods to produce the base hierarchical forecasts and we contrast any improvements from forecast combination across methods with the improvements of forecast combination across aggregation levels.
- We consider the case of forecast bias and we empirically bias-adjust the base forecast prior to hierarchical reconciliation. Bias adjustment of the base forecasts is expected to lead to less biased final reconciled forecasts but also to improve their accuracy.
- We explore the effect of seasonal shrinkage and selectively apply temporal hierarchies for forecasting based on the seasonal significance of the original series, as well as that of the rest of the temporal levels. Selectively applying temporal hierarchies (thus, avoiding excess seasonal shrinkage) can potentially improve forecasting accuracy by properly capturing the seasonality component.

The rest of the paper is organised as follows. Section 2 presents the background research and advances in hierarchical forecasting as well as the identified research gaps. In Section 3, we present the three proposed strategies to improve the performance of temporal hierarchies. In Section 4, we evaluate the proposed strategies by applying temporal hierarchical forecasting on a large collection of real series. The results are analysed on the basis of two error measures. Lastly, Section 5 concludes the study, presents its limitations and explores avenues for future research.

## 2 Background research

### 2.1 Hierarchical forecasting

**Fig 1:**
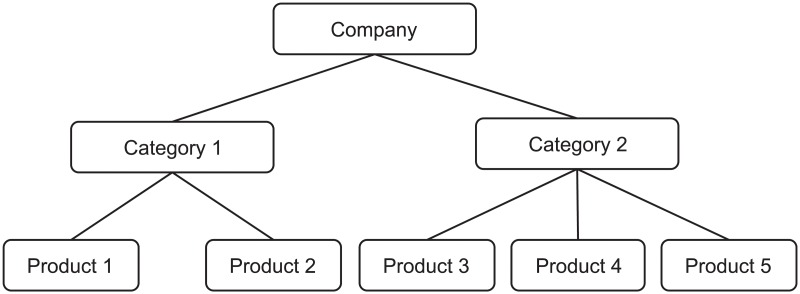
A cross-sectional hierarchy.

**Fig 2:**
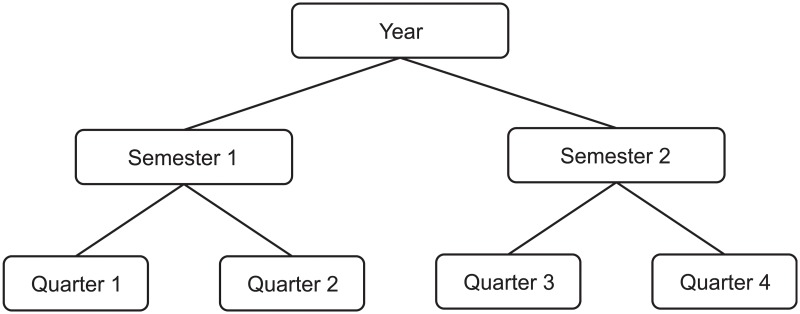
A temporal hierarchy.

Often, business data are arranged in hierarchical structures. Such structures decompose company units into different divisions, geographical areas and functions, in which case the structure is known as *cross-sectional hierarchies* [1, 4]. An example of a three-level cross-sectional hierarchy is depicted in Fig 1, where the sales of a company are disaggregated into two categories, with the first category consisting of two products and the second category consisting of three more products. Alternatively, hierarchical structures can be used to represent data depicted in different frequencies, in which case such structures are known as *temporal hierarchies* [3]. An example of a three-level temporal hierarchy is displayed in Fig 2, where the yearly data are disaggregated into two semesters and, consequently, into four quarters.

One property of organising the data in hierarchical structures is that the summation constrains hold for all the actual (observed) data. This means that the sum of the units of the products 1 and 2 in the cross-sectional hierarchy of Fig 1 equals to the units of the category 1. Similarly, the sum of the past data of all quarters within a year (Fig 2) equals to the respective yearly data. In fact, if one knows the values of the bottom-level data only, then it is trivial to derive the values of any other node. Assuming that the vector ***y***_*b*_ holds the bottom level data, then the data for all nodes within a hierarchy is given by ***y*** = ***Sy***_*b*_, where ***S*** is a summing matrix of order *m* × *n*, where *m* is the total number of nodes in the hierarchy and *n* is the number of nodes at the bottom level of the hierarchy. For the hierarchy depicted in Fig 1 (*m* = 8 and *n* = 5), the summing matrix is

$$\begin{array}{c} S=[\begin{array}{ccccc} 1 & 1 & 1 & 1 & 1\\ 1 & 1 & 0 & 0 & 0\\ 0 & 0 & 1 & 1 & 1\\ 1 & 0 & 0 & 0 & 0\\ 0 & 1 & 0 & 0 & 0\\ 0 & 0 & 1 & 0 & 0\\ 0 & 0 & 0 & 1 & 0\\ 0 & 0 & 0 & 0 & 1 \end{array}]. \end{array}$$ (1)

However, the summation constrain does not hold in the case of the forecasts that have been produced independently for each level. A simple solution would be to use the forecasts that have been produced on a single level and then derive (either by summation or by appropriate disaggregation) the forecasts of all other nodes, without forecasting them directly. To this direction, early studies on cross-sectional hierarchical forecasting compared approaches such as bottom-up, top-down and middle-out [6–10]. A disadvantage of using a single hierarchical level to compute forecasts is that data on a particular level might be too noisy or too aggregated to provide useful information for forecasting purposes. Also, focusing on one aggregation level does not allow for the full exploitation of the information provided from hierarchical structures. Studies comparing bottom-up with top-down approaches have been inconclusive with regards to the superiority of the one over the other. Interestingly, until quite recently a ‘bottom-up’ approach was considered the de facto approach in the temporal dimension, with forecasts being produced at the frequency in which the data have been collected.

A better solution would be to produce forecasts at all hierarchical levels and suitably combine them so that the forecasts across the various aggregation levels are *coherent*. Combining point forecasts across different cross-sectional [1, 2, 4, 11, 12], temporal [3, 13–19] as well as cross-temporal [20–22] aggregation levels has been extensively studied in the literature. More recently, studies have examined the case of probabilistic and density hierarchical forecasts as opposed to point forecasts [5, 23]. Two important insights from this stream of research is that (i) forecast accuracy improves if forecasts from different aggregation levels are suitably combined and (ii) coherent forecasts across aggregation levels allow for aligned decision making, both in terms of functions within a company (operations versus strategy) and horizons (short and long-term).

The coherent forecasts at all nodes of the hierarchy can be calculated as $\tilde{y}=S(S^{\prime }W^{-1}S{)}^{-1}S^{\prime }W^{-1}\hat{y}$, where $\hat{y}$ are the base (non-coherent) forecasts that have been produced independently at each node, ***S*** is the summing matrix and ***W*** is the covariance matrix of the base forecast errors. One challenge with this calculation is the estimation of ***W***. Earlier studies [1, 4] made a simplifying assumption about the in-sample forecasts errors, setting ***W*** = ***I***, thus avoiding its estimation. Effectively, the coherent forecasts were calculated assuming ordinary least squares (OLS) estimator. Later, [2] showed that ***W*** is unidentifiable and examined the performance of weighted least squares (WLS) via different estimators, including variance scaling and a trace minimisation approach. A simple estimator for ***W*** was proposed by [3], who suggested a structural scaling so that ***W*** = *diag*(***S*1**), where **1** is a unit column vector of size *n* (the number of nodes at the bottom level of the hierarchy). This estimator is easy to use as it depends only on the data structure while it is independent of the data and the forecasting methods. Moreover, its application showed superior performance on the temporal case compared to other variance-based estimators [3].

### 2.2 Research gaps

Regardless of the choice of estimator, in order for the coherent forecasts, $\tilde{y}$, to be unbiased, the base forecasts, $\hat{y}$, must also be unbiased. However, previous studies took for granted the unbiasedness of the base forecasts. In this study, we examine the effects of biased and unbiased base forecasts on hierarchical forecast reconciliation and propose two simple bias-adjustment approaches to further enhance the improvements on forecast accuracy as a result of hierarchical reconciliation.

Furthermore, previous studies examined the problem of hierarchical reconciliation by combining forecasts across hierarchical aggregation levels that have been produced using just one forecasting method per node. However, the forecasting literature has suggested that combination across different forecasting methods can also improve forecast accuracy and minimise uncertainty [24–27] even if simple equal-weighted combinations are considered [28] potentially by excluding extreme values [29]. We re-evaluate the performance of hierarchical forecasting by adding the *forecasting method* dimension, to the cross-sectional and temporal dimensions. Effectively, we suggest that the base forecasts should not be derived from a single method but a combination of methods and we empirically investigate the value-added of this additional level of combination.

Finally, we propose selectively applying temporal hierarchies to avoid damping the seasonal component of the series, a strategy that help us deal with the undesirable effect of averaging both seasonal and non-seasonal forecasts across different temporal levels. The study of [20] has shown that seasonally adjusting the series before applying temporal hierarchies mitigates the effect of seasonal shrinkage, leading to more reasonable as well as more accurate forecasts by exploiting the benefits of temporal aggregation while omitting its drawbacks. However, adjusting the data beforehand limits the potential of temporal aggregation in a sense that, if the forecasts of the rest of the levels are also seasonal, there is no reason discarding them a-priory. In this respect, we suggest selecting traditional forecasting over temporal hierarchies only if there is enough evidence that the aggregated data are non-seasonal, while the original data are strongly seasonal.

## 3 Strategies for improving the performance of temporal hierarchies

The present study proposes three strategies for improving the performance of temporal hierarchies, that is (i) combining forecasts of different methods for generating the base forecasts at each level of the hierarchy, (ii) adjusting the base forecasts so that their bias is mitigated and (iii) avoiding shrinking seasonality. Note that the proposed strategies can be applied either separately or simultaneously. Moreover, these are complements to the method considered for reconciling the base forecasts, being completely independent from each other. In this study, we apply these three strategies together with the WLS estimator which implements structural scaling (WLS_*S*_) for reconciling the hierarchy, given its reported superiority over other alternatives, such as the hierarchy variance (WLS_*H*_) and the series variance (WLS_*V*_) estimators [3].

### 3.1 Combining forecasts from multiple methods

Combining forecasts has long been considered an effective strategy for improving forecasting performance [30–32]. Many studies have investigated the reasons behind its success, proposing interesting alternatives for implementing it in practice [33]. Moreover, the forecast combination puzzle [34] has been both explored [35, 36], providing useful insights. More recently, the results of the M4 Competition [37] reconfirmed the benefits of combining and highlighted some innovative ways for its exploitation.

The superiority of combining forecasts generated by various forecasting methods over individual ones indicates that no single method can adequately capture the patterns of any possible type of series. Each method is good at capturing different time series components, meaning that combinations allow us to effectively capture greater and more complex patterns of series. Thus, combining accurate, as well as diverse, uncorrelated forecasts, results to far more robust extrapolation solutions that extract valuable information from the data originally provided, canceling at the same time the errors of the individual methods considered [38].

Temporal hierarchies imply one smart way of combining forecasts. Different information is extracted at each aggregation level where the signals of various time series components are either strengthened or attenuated [14]. Moreover, as the base forecasts are combined, both modelling and data uncertainty is mitigated, leading to more accurate and robust results [3].

However, when a single forecasting method is considered across all aggregation levels, it is still possible for some of the time series components to be inadequately captured. On the other hand, if various forecasts were to be averaged at each level, the benefits of combining would be further emphasised. This becomes evident if we observe that by introducing *n* forecasts per aggregation level, the base forecasts used for computing the reconciled ones grow *n* times in numbers. Thus, utilising multiple forecasting methods for generating the base forecasts not only helps us extract more information from the series, but also indirectly enhances the beneficial effects of combining by expanding the number and heterogeneity of the forecasts used by the reconciliation framework.

Undoubtedly, there are many ways for combining forecasts of different forecasting methods. The most common way is to average them using equal weights or other simple operators like the median, the mode or the trimmed mean [39, 40]. Such combinations are easy to apply and display minimum computational requirements as the weights derive from the forecasts alone, having zero dependencies from the historical performance of the examined methods. Moreover, it has been repeatedly shown that simple combinations provide similar or even more accurate results than complex approaches, being also more robust in nature [41]. This was also the case in the M4 Competition where the top performing combination method [42], determining the weights through a complex machine learning algorithm, did not provide much better forecasts than a much simpler approach that considered the median of four standard statistical methods [29].

In this regard, this first strategy involves the estimation of the simple, equally weighted average of two, diverse and easy to compute base forecasts, i.e., the utilisation of the most efficient combination scheme reported in the literature. Of course, it is up to the decision maker to select a more complex combination scheme than the one described above. We should note, however, that the forecasts produced by this scheme are basically the input of the method that will be considered for reconciling them, also implying a combination. As a result, any difference reported between such combination schemes are expected to be negligible. Our choice can be further supported by taking into account the trade-off between computational requirements and accuracy improvements [43]. In order for an improvement to be meaningful, it must come with a reasonably low additional computational cost. Temporal aggregation is a computationally expensive process on its own, meaning that further increasing its cost for achieving minor accuracy improvements is probably impractical. This is relevant both when dealing with long, high-frequency series, where generating forecasts individually is computationally expensive, and short, low-frequency ones, where generating forecasts individually is computationally cheap but possibly comparably expensive if considered for numerous series [44].

Finally, we should note that the reconciliation methods found in the literature adjust the base forecasts using linear operators. In this respect, reconciling the average forecasts of methods A and B is practically equivalent to reconciling the forecasts of method A and method B individually and then averaging them. For reasons of simplicity, in this study we adopt the first approach which requires reconciling the forecasts just once.

### 3.2 Bias adjustments

A key factor for optimally reconciling the base forecasts of a hierarchy is to estimate their covariance matrix. For some time, performing such a task was avoided and simpler alternatives, such as the ordinary least squares (OLS), were used instead to approximate its results [1]. However, it was recently shown that the covariance matrix is not identifiable and that an estimator which minimises the variances of the reconciled forecast errors can be exploited for constructing unbiased reconciled forecasts [2]. Yet, in order for the reconciled forecasts to be unbiased, the base forecasts must be unbiased as well. Undoubtedly, this assumption is rarely fulfilled in practice, meaning that the reconciliation performed might be far from optimal.

We will not pretend that generating unbiased forecasts is a trivial task, nor do we believe that any forecasting method is capable of producing perfectly unbiased results. Nevertheless, in our point of view, mitigating the bias of the base forecasts through appropriate adjustments is a promising strategy for improving the performance of temporal hierarchies by better fulfilling the assumptions made by the reconciliation frameworks utilised.

Producing unbiased base forecasts may also come with additional benefits, such as improved forecasting accuracy. The bias-variance decomposition, according to which the Mean Square Error (MSE) is decomposed into a bias (*B*) and a variance (*V*) term, is a fundamental concept in forecasting [45], expressed as

$$MSE=B^{2}+V.$$ (2)

The bias represents the consistent distance observed between the forecasts and the true values. The larger the distance, the higher the bias of the forecasts and vice-versa. On the other hand, the variance represents the variation of the forecasts around their mean. Similarly, as the variation of the forecasts increases for different realisations of the error terms, the variance becomes higher and vice-versa.

Observe that it is possible for two completely different forecasts, the first one characterised by little bias but much variance and the second one by much bias but little variance, to display exactly the same level of accuracy. However, given two forecasts of the same variance, the less biased forecast will be also the more accurate one. Thus, in our case, for any base forecast provided, we can assume that there is an offset value, i.e. a bias, which deteriorates forecasting accuracy and if effectively adjusted can lead to more accurate results, although maintaining its original variance.

Unfortunately, the research done in the field of forecasting for implementing bias adjustments is rather limited. Most of the research focuses on judgmental adjustments [46, 47] which are not applicable in our case, or model-specific parameter corrections [48] that can not be adopted within generalised forecasting frameworks. However, [49] describe some practical alternatives, indicating some simple, yet effective approaches for performing this task. In the present study we expand these alternatives by considering both additive and multiplicative bias adjustments, which are defined as

$${\hat{y}}_{n+h}^{a}={\hat{y}}_{n+h}+\frac{1}{n}\sum_{i=1}^{n}y_{i}-{\hat{y}}_{i}$$ (3)

$${\hat{y}}_{n+h}^{m}={\hat{y}}_{n+h}\times \frac{1}{n}\sum_{i=1}^{n}\frac{y_{i}}{{\hat{y}}_{i}},$$ (4)

where *y*_*i*_ is the actual values of series *y* at point *i*, ${\hat{y}}_{i}$ is the respective forecast, *h* is the forecasting horizon, *n* is the number of the data points available in-sample and ${\hat{y}}_{i}^{a}$ and ${\hat{y}}_{i}^{m}$ are the additively and multiplicatively adjusted forecasts respectively.

Note that in both cases, the out-of-sample forecasts are adjusted based on the average bias observed in-sample. Thus, the main assumption made by the proposed strategy is that the extent of bias reported while fitting the forecasting method will remain the same when extrapolating the series in the future. The only difference between the two approaches is that additive adjustments assume that the bias is a constant, while the multiplicative ones that the bias is a ratio.

Observe also that the factors used for adjusting the original forecasts can be estimated using other operators than the mean. For instance, the median or the mode could be used to mitigate the effect of extreme residuals and provide more indicative results.

### 3.3 Avoiding seasonal shrinkage

When applying temporal hierarchies on time series, the produced forecasts tend to be much smoother than the original data as aggregating across multiple temporal levels acts as a moving average filter [20, 50]. This can highly affect the component of seasonality which, in contrast to those of level and trend, can only be estimated only for a particular subset of aggregation levels. For instance, monthly series are predicted across six different levels (annual, semi-annual, four-monthly, quarterly, bi-monthly and monthly) for which the forecasts of the highest level (yearly) will be definitely non-seasonal. Taking also into consideration that seasonality is less likely to be observed at high aggregation levels, where the original curvatures are shrank, it is possible for more levels to derive non-seasonal forecasts, leading to damped seasonality.

In general, seasonality shrinkage is considered an effective practice for improving forecasting accuracy, especially when dealing with short series and noisy data where estimation of seasonality involves greater uncertainty [51, 52]. The same stands for temporal hierarchies where different types of seasonality may be identified across different aggregation levels, providing a better representation of seasonal variations [3]. However, the beneficial effect of shrinkage may not always stand in practice, particularly when the original data are strongly seasonal but the aggregated ones are not. In such cases, unnecessarily damping the seasonal component will lead to inaccurate results.

In order to deal with this problem, this last strategy involves an heuristic rule for avoiding seasonal shrinkage, as follows:

- The existence of seasonality is examined across all aggregation levels.
- If the series is not seasonal in the original frequency, then temporal hierarchies are applied.
- If the series is seasonal in the original frequency, then temporal hierarchies are applied only if at least half of the aggregated levels are seasonal. Otherwise, the base forecasts of the lowest level of the hierarchy are used for extrapolation, being reconciled for the rest of the levels through the bottom-up method.

There are a lot of options available in the literature for testing whether a series is seasonal or not. The simplest solution would probably be to perform a seasonality test based on the auto-correlation significance of the *m*^th^ term of the ACF, where *m* is the frequency of the series. This approach has been applied to adjust the data for seasonality in the theta method [53, 54] as well as in the recent M4 forecasting competition [37]. According to this test, a series is identified as seasonal at the (1 − *α*)% confidence level when

$$|{\text{ACF}}_{m}|>q_{1-\alpha /2}\sqrt{\frac{1+2\sum_{i=1}^{m-1}{{\text{ACF}}_{i}}^{2}}{n}},$$ (5)

where *n* is the number of observations in the series (in-sample size), ACF_*k*_ is the autocorrelation function at lag *k* and *q* is the quantile function of the normal distribution. We opt for a confidence level of 90% (*α* = 0.1, *q*_0.95_ = 1.645), in line with past research (see the references above).

An alternative would be to fit different models to the series, both seasonal and non-seasonal ones, and decide whether the data is seasonal based on the model that reported the best in-sample performance. For example, this is a trivial task for the case of exponential smoothing family of models which assumes no, additive or multiplicative seasonality [55], as well as the AutoRegressive Integrated Moving Average (ARIMA) models which include seasonal differencing or not [56]. Thus, using forecasting models as proxies for identifying significant seasonality, relevant conclusions can be reached.

In order to mitigate the uncertainty present when testing for seasonality, we proceed by combining both the approaches presented above, deciding based on the majority of the reported results. Thus, for each series, the ACF test is performed and the “optimal” exponential smoothing and ARIMA models are identified. Then, in order for a series to be classified as seasonal, at least two of the following must be true:

- Eq 5 is true.
- The identified “optimal” exponential smoothing model has a seasonal component (either additive or multiplicative).
- The identified “optimal” ARIMA model involves seasonal differencing (the order of differencing is irrelevant).

Note that each of the criteria used above is good for capturing a different type of seasonality, meaning that combining their results makes selection more generic and robust. For example, the ACF test examines the existence of a deterministic seasonality, exponential smoothing assumes either an additive or multiplicative process for a linear or exponential change in the seasonality, while ARIMA pinpoints a stochastic seasonal component.

## 4 Empirical evaluation

### 4.1 Design

To empirically evaluate the three strategies proposed in Section 3, we use the monthly time series from the two most cited forecasting competitions, the M [57] and the M3 [58]. The M competition involves 617 monthly series, while the M3 1,428, resulting into a test sample that consists of 2,045 series in total. From the competitions’ datasets we decided to use only the series of the highest frequency, excluding the yearly and quarterly ones, given that the beneficial effects of temporal aggregation are more likely to be observed when information from multiple aggregation levels is combined [3].

In order to enable direct comparisons with published results, we maintain the in-sample of the series unchanged and use its whole length for training the forecasting methods. The forecasting horizon at the monthly frequency (first aggregation level) is *h*_1_ = 18, which corresponds to what was requested by the organisers of the competitions. We construct temporal hierarchies, as proposed by [3], by aggregating the monthly series to bi-monthly, quarterly, four-monthly, semi-annual and annual levels. Each temporal hierarchy covers 12 periods in the monthly frequency. In order to cover all 18-months of the forecasting horizon in the monthly frequency, we produce forecasts for two temporal hierarchies ahead (24 monthly forecasts, equal to 2 years). Finally, we use the first *h*_*k*_ = ⌊*h*_1_/*k*⌋ periods of each frequency to evaluate the performance, where ⌊⋅⌋ is the floor function and *k* is the aggregation level (*k* = 3 for quarterly data).

For each series and at each aggregation level, we independently generate base forecasts using the automated algorithms for ExponenTial Smoothing (ETS) [55] and ARIMA [56] models, as well as their simple, equally weighted COMBination (COMB). The first two methods, ETS and ARIMA, are widely utilised in the forecasting literature during the last few years, especially in studies focused on hierarchical forecasting, and are capable of providing the “best” exponential smoothing and ARIMA model, respectively, indicated through information criteria. The last method, COMB, is introduced to assess the effect of the first strategy proposed in this paper for improving the performance of hierarchical forecasting through forecast combinations. All forecasts are generated using the *forecast* package for R [59].

The base forecasts are first reconciled by applying the Bottom-Up (BU) method. In this scenario, the base forecasts estimated at the lowest level of the hierarchy (monthly data) are aggregated to provide forecasts for the rest of the temporal levels. Given the simplicity of the method and the fact that it does not consider information from frequencies other than the one originally available, BU forms a natural benchmark that allows fair comparisons with the strategies proposed in this study.

Base forecasts are then adjusted to mitigate bias. Both ADDitive (ADD) and MULtiplicative (MUL) bias adjustments are considered using the median operator. Note that similar results are obtained when using the mean operator and, therefore, these are not presented for reasons of brevity. Another reason of using the median operator instead of the mean, is that the latter reported slightly less accurate results for the case of the additive adjustments, in contrast to the former which worked equally well both for additive and multiplicative adjustments. We proceed by generating reconciled forecasts using the WLS_*S*_ estimator.

Finally, we evaluate the last strategy proposed in this study by selectively applying temporal hierarchies to avoid excessive seasonality damping, as described in Section 3.3. The automatic selection algorithms of ETS and ARIMA utilised for generating the base forecasts are also exploited for examining the existence of seasonality, together with the traditional ACF test. Based on this strategy, temporal hierarchies are not applied to 150 out of the 2045 series in the sample.

The forecasts are evaluated both in term of forecasting accuracy (closeness of actual values and forecasts) and bias (consistent differences between actual values and generated forecasts). We use the Mean Absolute Scaled Error (MASE) [60] and the Absolute value of the Scaled Mean Error (ASME) [16] which permit averaging forecasting performance across time series of different scales. For both MASE and ASME, lower values are better. The MASE and the ASME are defined as

$$\text{MASE}=\frac{1}{h}\frac{\sum_{t=n+1}^{n+h}|y_{t}-{\hat{y}}_{t}|}{\frac{1}{n-m}\sum_{i=m+1}^{n}\left|y_{t}-y_{t-m}\right|},$$ (6)

$$\text{ASME}=\frac{1}{h}\frac{\left|\sum_{i=n+1}^{n+h}y_{t}-{\hat{y}}_{t}\right|}{\frac{1}{n}\sum_{i=1}^{n}y_{i}},$$ (7)

where *y*_*t*_ is the actual value of series *Y* at point *t*, ${\hat{y}}_{t}$ is the respective forecast of the method being evaluated, *h* is the forecasting horizon at each frequency, *n* is the number of the data points available in-sample and *m* is the data frequency, i.e., 12 for monthly, 6 for bi-monthly, 4 for quarterly, 3 for four-monthly, 2 for semi-annual and 1 for annual levels. Note that depending on the original length of the series and the temporal level examined, different *n* and *h* values are considered. The results are summarised both for each aggregation level and in total by averaging the performance computed for the six individual aggregation levels.

Finally, we would like to note that the results based on MASE are directly comparable with those based on the symmetric Mean Absolute Percentage Error (sMAPE), the measure originally used in the M3 Competition to evaluate forecasting accuracy and a common choice in the forecasting literature. Given the similarity of the results, as well as the drawbacks of sMAPE [61], we proceed by reporting only the MASE results.

### 4.2 Results

Tables 1 and 2 summarise the results for the MASE and ASME measures, respectively. The first row of each method (ETS, ARIMA and COMB) presents the results of the BU reconciliation, that is used as benchmarks for measuring the effect of the strategies proposed in this paper for improving the performance of hierarchical forecasting: (i) Combining forecasts of multiple methods, (ii) applying bias adjustments and (iii) avoiding seasonal shrinkage. The following rows present the performance of the WLS_*S*_ reconciled forecasts. Each column of the tables represents an aggregation level, while the last column provides the average performance across all aggregation levels. Entries in bold highlight the most accurate forecasting approach per case.

**Table 1:** Forecasting performance in terms of accuracy for the 2,045 monthly series of the M and M3 competitions. The results are averaged across all series, both per aggregation level and across all levels. Additive (ADD), multiplicative (MUL) or none bias adjustments were considered. Results are also reported when temporal hierarchical forecasting is applied selectively to avoid seasonal shrinkage.

Forecasting method	Reconciliation method	Bias adjustment	Avoiding seasonal shrinkage	Annual	Semi annual	Four monthly	Quarterly	Bi monthly	Monthly	Average
ETS	BU	None	No	1.005	1.051	0.961	0.983	0.962	0.928	0.982
	WLS_*S*_	None	No	0.959	1.015	0.931	0.956	0.937	0.909	0.951
	WLS_*S*_	MUL	No	0.936	0.999	0.915	0.941	0.922	0.896	0.935
	WLS_*S*_	ADD	No	0.935	0.998	0.914	0.940	0.921	0.895	0.934
	WLS_*S*_	None	Yes	0.945	1.007	0.922	0.948	0.929	0.902	0.942
	WLS_*S*_	MUL	Yes	0.921	0.991	0.907	0.933	0.915	0.889	0.926
	WLS_*S*_	ADD	Yes	0.920	0.990	0.905	0.932	0.914	0.888	0.925
ARIMA	BU	None	No	1.025	1.080	0.981	1.009	0.982	0.944	1.004
	WLS_*S*_	None	No	0.970	1.025	0.938	0.962	0.938	0.908	0.957
	WLS_*S*_	MUL	No	0.960	1.017	0.932	0.955	0.932	0.903	0.950
	WLS_*S*_	ADD	No	0.958	1.016	0.930	0.954	0.931	0.902	0.948
	WLS_*S*_	None	Yes	0.966	1.024	0.937	0.961	0.938	0.907	0.955
	WLS_*S*_	MUL	Yes	0.957	1.017	0.932	0.955	0.932	0.903	0.949
	WLS_*S*_	ADD	Yes	0.955	1.015	0.930	0.954	0.931	0.902	0.948
COMB	BU	None	No	0.978	1.028	0.936	0.961	0.938	0.905	0.958
	WLS_*S*_	None	No	0.948	1.000	0.916	0.940	0.918	0.889	0.935
	WLS_*S*_	MUL	No	0.930	0.988	0.906	0.929	0.908	0.880	0.924
	WLS_*S*_	ADD	No	0.929	0.988	0.905	0.929	0.908	0.880	0.923
	WLS_*S*_	None	Yes	0.937	0.994	0.909	0.933	0.913	0.884	0.928
	WLS_*S*_	MUL	Yes	0.919	0.982	0.899	0.923	0.903	0.876	0.917
	WLS_*S*_	ADD	Yes	**0.918**	**0.982**	**0.899**	**0.923**	**0.903**	**0.876**	**0.917**

The most accurate forecasting approach per temporal aggregation level is highlighted in bold.

**Table 2:** Forecasting performance in terms of bias for the 2,045 monthly series of the M and M3 competitions. The results are averaged across all series, both per aggregation level and across all levels. Additive (ADD), multiplicative (MUL) or none bias adjustments were considered. Results are also reported when temporal hierarchical forecasting is applied selectively to avoid seasonal shrinkage.

Forecasting method	Reconciliation method	Bias adjustment	Avoiding seasonal shrinkage	Annual	Semi annual	Four monthly	Quarterly	Bi monthly	Monthly	Average
ETS	BU	None	No	0.092	0.111	0.105	0.111	0.111	0.111	0.107
	WLS_*S*_	None	No	0.086	0.103	0.098	0.104	0.104	0.104	0.100
	WLS_*S*_	MUL	No	0.085	0.103	0.097	0.103	0.103	0.103	0.099
	WLS_*S*_	ADD	No	0.084	0.102	0.096	0.102	0.102	0.102	0.098
	WLS_*S*_	None	Yes	0.085	0.103	0.098	0.103	0.103	0.103	0.099
	WLS_*S*_	MUL	Yes	0.085	0.102	0.097	0.103	0.103	0.103	0.099
	WLS_*S*_	ADD	Yes	**0.084**	**0.101**	**0.096**	**0.102**	**0.102**	**0.102**	**0.098**
ARIMA	BU	None	No	0.097	0.118	0.111	0.118	0.118	0.119	0.114
	WLS_*S*_	None	No	0.090	0.109	0.103	0.109	0.109	0.109	0.105
	WLS_*S*_	MUL	No	0.090	0.107	0.102	0.108	0.108	0.108	0.104
	WLS_*S*_	ADD	No	0.089	0.107	0.101	0.107	0.107	0.108	0.103
	WLS_*S*_	None	Yes	0.090	0.109	0.103	0.109	0.109	0.109	0.105
	WLS_*S*_	MUL	Yes	0.089	0.107	0.102	0.108	0.108	0.108	0.104
	WLS_*S*_	ADD	Yes	0.089	0.107	0.101	0.107	0.108	0.108	0.103
COMB	BU	None	No	0.090	0.109	0.103	0.109	0.109	0.109	0.105
	WLS_*S*_	None	No	0.086	0.104	0.098	0.104	0.104	0.104	0.100
	WLS_*S*_	MUL	No	0.085	0.103	0.097	0.103	0.103	0.103	0.099
	WLS_*S*_	ADD	No	0.085	0.102	0.097	0.103	0.103	0.103	0.099
	WLS_*S*_	None	Yes	0.086	0.103	0.098	0.103	0.103	0.103	0.099
	WLS_*S*_	MUL	Yes	0.085	0.102	0.097	0.102	0.103	0.103	0.099
	WLS_*S*_	ADD	Yes	0.084	0.102	0.096	0.102	0.102	0.102	0.098

The most unbiased forecasting approach per temporal aggregation level is highlighted in bold.

**Fig 3:**
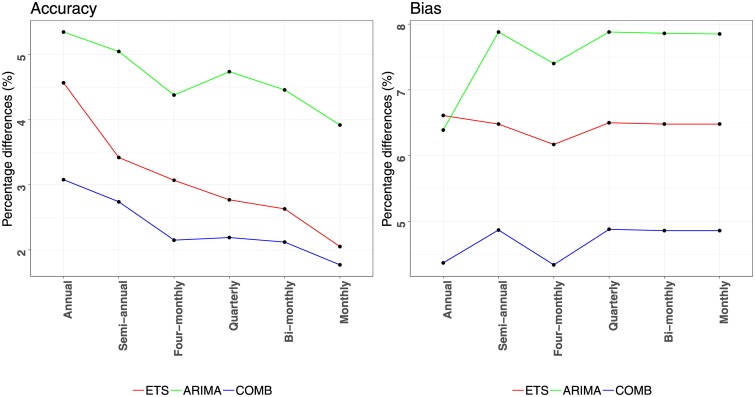
Forecasting performance improvements reported for applying WLS_*S*_ reconciliation instead of BU. The improvements (percentage differences) are estimated per aggregation level for the ETS (red), ARIMA (green) and COMB (blue) methods, both in terms of accuracy (MASE) and bias (ASME).

Before proceeding with the evaluation of the three strategies proposed in this paper, we should first highlight that these are complements to the WLS_*S*_ temporal aggregation, a framework which has been proven to significantly improve the base forecasts provided to it as input. Thus, further improving the performance of the framework becomes a promising, yet challenging task. This becomes evident in Fig 3 which presents the percentage differences between the WLS_*S*_ reconciled forecasts and the base ones. The improvements are provided per aggregation level for the three forecasting methods examined in this study, both in terms of accuracy and bias. Observe that the accuracy of all the methods is enhanced at all aggregation levels, ranging from 1.8% to 5.4%. The improvements are higher at the highest temporal levels and are gradually declined as the frequency of the data increases. This is due to the limited sample of the low frequency series, which become more predictable as information from high frequency series becomes available. On the other hand, high frequency series are benefited by incorporating the trend component of the data which is easier to capture at high aggregation levels. Significant improvements are also observed for the case of bias, ranging from 4.3% to 7.9%. However, in contrast to accuracy, bias improvements are relatively stable across all aggregation levels, indicating that combining information from multiple levels helps us stabilise the bias globally. It is also notable that the improvements of temporal aggregation are greater for the less accurate/more biased methods. Thus, although its utilisation is always recommended, accurate/unbiased base forecasts are less likely to be substantially improved than inaccurate/biased ones.

**Fig 4:**
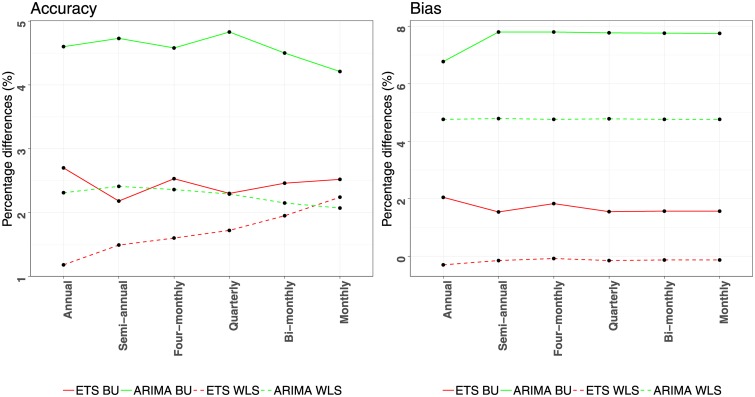
Forecasting performance improvements reported for using a combination of forecasts instead of individual ones. The improvements (percentage differences) are estimated per aggregation level, both in terms of accuracy (MASE) and bias (ASME). Comparisons of COMB are presented independently for ETS (red) and ARIMA (green), both for applying the BU (solid) and WLS_*S*_ (dashed) reconciling methods.

The results presented in Tables 1 and 2 demonstrate that forecasting using COMB results in significant forecast accuracy and bias improvements for all aggregation levels over both ETS and ARIMA forecasts. The improvements are larger for ARIMA compared to ETS as ARIMA provides less accurate and more biased base forecasts than ETS. Moreover, the improvements reported are relatively constant across the six aggregation levels, indicating that COMB leads to consistent better forecasts regardless the data frequency examined. Observe also that COMB is beneficial both for the BU and WLS_*S*_ reconciliation methods, verifying that combining forecasts from different methods is a strategy that can effectively enhance temporal hierarchies no matter what kind of reconciliation methods are being used. Thus, we conclude that combining forecasts from different aggregation levels and forecasting methods concurrently, results in more accurate and less biased forecasts than the base forecasts generated for each aggregation level separately or the reconciled forecasts generated using single forecasting methods. The improvements of using COMB instead of ETS or ARIMA individually are visualised in Fig 4 and are presented per aggregation level for the BU and WLS_*S*_ reconciliation methods, both in terms of accuracy and bias. As seen, the accuracy improvements range from 1.2% to 4.8%, with the bias improvements reaching up to 7.8%. Note that combining forecasts of different forecasting methods displays similar or even greater percentage improvements over combining forecasts of different aggregation levels. Therefore, the first strategy proposed in Section 3.1 adds considerable value to temporal hierarchies.

**Fig 5:**
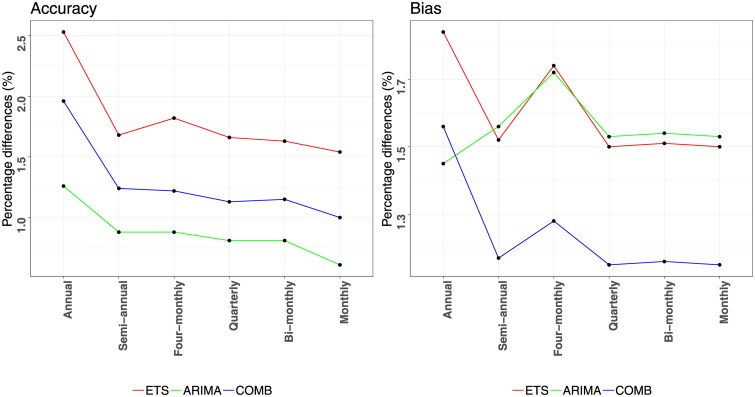
Forecasting performance improvements reported for applying additive bias adjustments instead of original WLS_*S*_ reconciliation. The improvements (percentage differences) are estimated per aggregation level for the ETS (red), ARIMA (green) and COMB (blue) methods, both in terms of accuracy (MASE) and bias (ASME). A similar graph is obtained for the case of multiplicative bias adjustments.

Regarding the effect of bias adjustments, the results show that modifying the base forecasts to mitigate bias, either in an additive or in a multiplicative way, also leads to improvements in forecasting performance. The percentage differences for the additive bias adjustments are visualised in Fig 5 (the percentage differences for multiplicative adjustments are very similar). These range from 0.6% to 2.5% for MASE and from 1.2% to 1.8% for ASME. Hence, we conclude that applying bias adjustments to the base forecasts before reconciling them results in forecasts that are not only less biased, but also more accurate. Observe that the accuracy improvements are larger at the higher aggregation levels, especially the annual level, while the bias improvements are relatively stable. Therefore, the second strategy proposed in Section 3.2 is also shown to considerably enhance the forecasting performance of temporal hierarchies.

**Fig 6:**
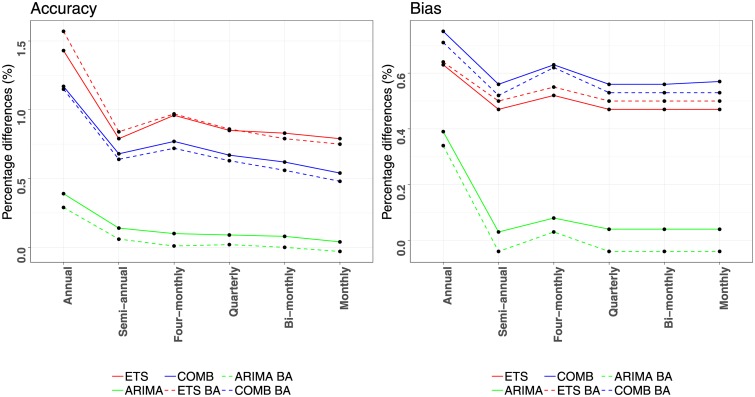
Forecasting performance improvements reported for selectively applying temporal hierarchies to avoid seasonal shrinkage. The improvements (percentage differences) are estimated per aggregation level for the ETS (red), ARIMA (green) and COMB (blue) methods, both in terms of accuracy (MASE) and bias (ASME). The WLS_*S*_ estimator is used for reconciliation, either considering additive bias adjustments “BA” (dashed) or not (solid). A similar graph is obtained for the case of multiplicative bias adjustments.

The results of Tables 1 and 2 indicate that the last strategy proposed in Section 3 also has a positive effect on forecasting accuracy and bias, improving further the performance of temporal hierarchies. This effect is visualised in Fig 6 which reports the percentage differences of original WLS_*S*_ and selective application of temporal hierarchies to avoid seasonal shrinkage, either considering additive bias adjustments, or not. The improvements reach up to 1.6% and 0.8% for the case of MASE and ASME, respectively, being larger for the highest levels of the hierarchy.

Next, we attempt to disentangle the effects of the three strategies described in Section 3, assuming an additive bias-adjustment. Table 3 presents the average performance improvements when each strategy is applied separately as well as if two or more strategies are considered together. Improvements are measured based on the WLS_*S*_ hierarchical combinations when a single method (ETS or ARIMA) was used to produce the base forecasts. We observe that combining across methods (and not just aggregation levels) brings the largest improvements (around 2% on average), followed by the bias-adjustment strategy (on average 1.5% improvement). Performance improvements are similar for both forecast accuracy (MASE) and bias (ASME). We observe minimal interactions between the strategies, with the average improvement of multiple strategies applied at the same time being roughly equal to the sum of the average improvements when each strategy was considered separately.

**Table 3:** Average performance improvements when each strategy is applied separately or in conjuction with other strategies.

Combination of methods	Bias adjustment	Avoiding seasonal shrinkage	Accuracy improvement	Bias improvement
✔			1.98%	2.30%
	✔		1.34%	1.57%
		✔	0.54%	0.30%
✔	✔		3.24%	3.51%
✔		✔	2.71%	2.89%
	✔	✔	1.85%	1.84%
✔	✔	✔	3.91%	4.06%

To sum up, when the average performance of the forecasts is considered across all temporal levels, the combination of the three strategies proposed in this study lead to notable improvements, enhancing significantly the results of standard temporal aggregation methods. For example, for the case of ETS, the accuracy is improved by 6.6% and 3.6% when the BU or the WLS_*S*_ method is considered for reconciling the base forecasts, respectively. Accordingly, the bias of the methods is improved by 8% and 1.6%. The results are even better for the case of the ARIMA, where the accuracy is improved by 8.7% and 4.2% for the BU and the WLS_*S*_ method, respectively, while the bias by 13.6% and 6.5%.

**Fig 7:**
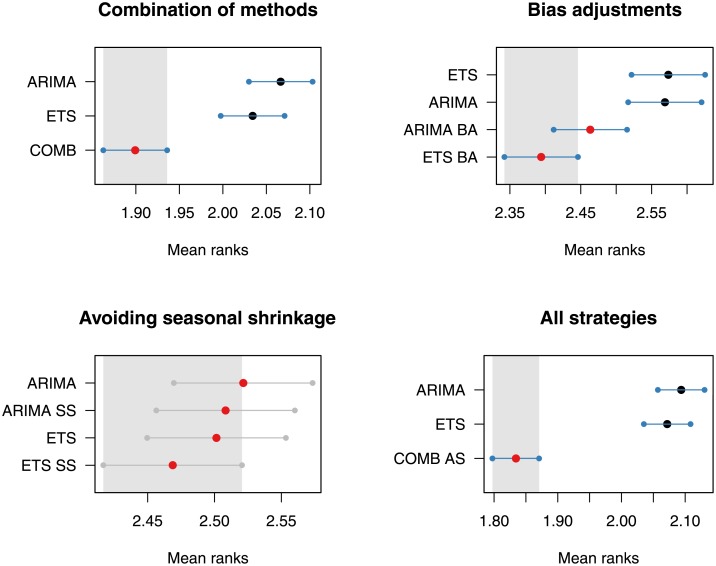
MCB significance tests for the three strategies considered. The analysis is done using the average MASE reported for each series across all temporal levels. In all cases, the WLS_*S*_ reconciliation method is considered. “COMB” is the combination of ETS and ARIMA forecasts (first strategy), “BA” is additive bias adjustments (second strategy), “SS” is the avoidance of seasonal shrinkage (third strategy) and “COMB AS” is the combination of the three strategies.

In order to verify that the improvements of the proposed strategies in terms of absolute forecasting performance are statistically significant, we apply Multiple Comparisons from the Best (MCB). MCB tests if the average ranking of each forecasting approach is significantly different than the others [62]. The null hypothesis of this test is rejected when the intervals of two approaches do not overlap, suggesting that their ranked performances are statistically different. The analysis is done using the average MASE reported for each series across all temporal levels. The three strategies are tested both separately and together against the two benchmarks (ETS and ARIMA) when the WLS_*S*_ reconciliation method is considered. The results are presented in Fig 7.

We observe that the combination of the three strategies (last panel) results in the best ranked performance which is also statistically different to that of the two benchmarks. It is also evident that combining across methods, as well as applying bias adjustments, leads to significantly better forecasts. On the other hand, although avoiding seasonal shrinkage leads to better forecasts compared to the two benchmarks, the differences reported are not statistically significant. Yet, given that this strategy is applied only to a limited number of series (7.3% of the examined series), it is difficult to draw clear conclusions about its true potential. Thus, we conclude that the first two strategies are effective in improving the forecasting performance of temporal hierarchies while more evidence is needed for evaluating the impact of the last strategy.

Finally, we would like to notice that the improvements reported by the proposed strategies do not significantly increase the computational cost of the forecasting process, as they utilise information that is directly provided during the estimation of the base forecasts, or imply minor, computationally cheap additional estimations (e.g., ACF test and bias estimation). For example, we have found that bias adjustments increase computational cost by about 0.6%, while the avoidance of seasonal shrinkage by less that 0.1%. Regarding the combination of the methods, the additional cost depends strongly on the computation time required for individually estimating the combined methods. Thus, given that ETS is much faster to compute than ARIMA, the additional cost for combining is about 1.3 and 4.7 times greater for the case of the ARIMA and the ETS methods, respectively. In this regard, these strategies transform temporal hierarchies into a truly efficient forecasting solution, capable of producing fast, yet highly accurate forecasts.

## 5 Conclusions

Temporal hierarchies have been proven an effective solution for improving the performance of traditional forecasting methods, supporting at the same time aligned decisions at different planning horizons. The accuracy, robustness, coherency and uncertainty mitigation they imply, have made them a popular forecasting framework, highlighting the potential benefits of multiple temporal aggregation in decision making.

Having examined the limitations of the existing framework, this study aims at further improving the performance of temporal hierarchies by considering three different strategies: (i) Combining forecasts of multiple methods, (ii) applying bias adjustments and (iii) selectively implementing temporal hierarchies to avoid seasonal shrinkage. These strategies can be applied either separately or simultaneously, being complements to the method considered for reconciling the base forecasts and completely independent from each other. Moreover, they are computationally cheap, utilising information which becomes directly available when generating the base forecasts or easy-to-compute derivatives of the forecasting process.

The results show that replacing base forecasts coming from a single method with combinations of multiple methods has a great positive impact on forecasting performance across all aggregation levels, especially when the individual methods used are inaccurate or biased. Similarly, mitigating the bias of the base forecasts leads to improved forecasting performance, being more significant at the higher levels of the hierarchy where historical data are typically scarce. Finally, selectively choosing between temporal hierarchies and traditional bottom-up modelling is also beneficial, allowing the better handling of highly-seasonal series for which the seasonal component is excessively damped by temporal hierarchies.

The strategies proposed in this study indicate that temporal hierarchies are a useful, yet generalised framework for combining forecasts of different temporal levels that can be further expanded to provide even more accurate and robust results. This work introduces such effective expansions, outlining some interesting ways towards that direction. Even if this study focuses on improving the performance of temporal hierarchies, the first two strategies presented could be equally considered for cross-sectional hierarchies. Future research could focus towards that direction. Finally, our work focuses on the forecasting performance of the point forecasts and it would be interesting to see if the results generalise for the case of probabilistic forecasts [5].
