# Supplementary material for: Improving the forecasting performance of temporal hierarchies
Source: PLoS One. 2019 Oct 3;14(10):e0223422. doi: 10.1371/journal.pone.0223422 (PMC6776316; doi:10.1371/journal.pone.0223422)
Supplement: Attachment — Submitted filename: PONE-D-19-19038 responses to comments.pdf [file pone.0223422.s001.pdf]

PONE-D-19-19038

Improving the forecasting performance of temporal hierarchies

Authors' responses to reviewer's comments

We would like to thank the AE and the reviewers for their very helpful and constructive comments and editing suggestions. Please find our point-to-point responses below.

Reviewer #1

The authors propose and discuss strategies to improve the performance of temporal hierarchical forecasting. They motivate their work by outlining drawbacks of current approaches and subsequently propose strategies to address them. The evaluation of the proposed methods on standard datasets underlines the effectiveness of the strategies.

I enjoyed reading the papers as it addresses an interesting and important topic and is generally well written. Overall, the paper is suitable for publication in PLOS ONE, but I have a few suggestions that would improve the paper.

1) Computational costs are mentioned in Section 3.1. How is this a (practically) relevant criteria for the rather short and monthly time series? How do the different strategies affect the total runtime? How much runtime do they add to the initial forecasts?

We clarified this issue as follows:

In Section 3.1 we mention that computational cost is relevant both when dealing with long, high-frequency series, where generating forecasts individually is computationally expensive, and short, low-frequency ones, where generating forecasts individually is computationally cheap but possibly comparably expensive if considered for numerous series.

In Section 4.2 we report the additional cost of the proposed strategies, providing an indication of their efficiency.

Finally, we support our arguments with two relevant references.

2) Where does Equation 5 come from? A more detailed explanation could be beneficial.

We now provide more details on the seasonality test based on autocorrelation test (three references have been also added), as follows:

This approach has been applied to adjust the data for seasonality in the theta method for forecasting [50,51] as well as in the recent M4 forecasting competition [37]. A series is identified as seasonal at the $(1-\alpha)$% confidence level when

$$|\mathrm{ACF}_m| > q_{1-\alpha/2}\sqrt{\frac{1+2\sum_{i=2}^{m-1}\mathrm{ACF}_i^{\,2}}{n}}$$

where n is the number of observations in the series (in-sample size), ACF_k is the autocorrelation function at lag k and q is the quantile function of the normal distribution. We opt for a confidence level of 90% (α = 0.1, $q_{0.95}$ = 1.645), in line with past research (see the references above).

3) Why is the third strategy called seasonal shrinkage (damping seasonality)? Is it not just an indicator when temporal hierarchical forecasting, which would shrink the seasonality, should not be applied?

This is a fair comment. We made several changes within the manuscript (including, abstract, introduction, methodology, results and conclusions) and we now present the third strategy as “selectively applying temporal hierarchies to avoid seasonal shrinkage”. Section 3.3 has been renamed as “Avoiding seasonal shrinkage”.

4) How does the temporal hierarchy which is considered for empirical evaluation look like? The fact that the forecast horizon covers 18 months (more than one year) and that the last 2 (4) predictions are excluded (in some cases) makes different hierarchies possible.

Thank you for this comment. We have revise the second paragraph of Section 4.1 accordingly:

The forecasting horizon at the monthly frequency (first aggregation level) is $h_1 = 18$, which corresponds to what was requested by the organizers of the competitions. We construct temporal hierarchies, as proposed by [3], by aggregating the monthly series to bi-monthly, quarterly, four-monthly, semi-annual and annual levels. Each temporal hierarchy covers 12 periods in the monthly frequency. In order to cover all 18-months of the forecasting horizon in the monthly frequency, we produce forecasts for two temporal hierarchies ahead (24 monthly forecasts, equal to 2 years). Finally, we use the first $h_k = \lfloor h_1 / k \rfloor$ periods of each frequency to evaluate the performance, where $\lfloor \diamond \rfloor$ is the floor function and k is the aggregation level ($k = 3$ for quarterly data).

5) Why is the mean not used for bias-adjustments as introduced in Equations 3+4? You can still mention that the results are similar using the median operator.

The main reason of using the median operator instead of the mean, is that the latter leads to slightly less accurate results for the case of the additive adjustments, in contrast to the former which works equally well for additive and multiplicative adjustments. Thus, when using the median, the same improvements are achieved regardless of the nature of the adjustment. Yet, once again, we note that the differences between the two approaches are insignificant, to the degree of 10^-2.

6) Why are the results not tested for statistical significance?

At the end of Section 4 we have included an analysis testing for statistical significance. We use the Multiple Comparisons from the Best (MCB) test that compares if the average (across series) ranking of each forecasting approach is significantly different than the others. The proposed strategies are tested both individually and together. The results of the tests verify our conclusions.

7) Are the results comparable for SMAPE, which is also a frequently used accuracy measure?

At the end of Section 4.1 we mention that our results according to MASE are directly comparable with those of sMAPE. Given the similarity of the results, as well as the drawbacks of sMAPE, we proceed by reporting only those of MASE.

8) While the result section contains all relevant information and the content is also structured, the structure can be improved. I would recommend making a better effort to disentangle the effects of the different strategies. This would also help to emphasize the answer to the initial research question: "What affects the performance?" All strategies improve the results, but how do they interact? Which is the most important strategy?

We thank the reviewer for this very useful comment. As suggested, we have added a paragraph and a table (as below) to better explain the improvements achieved from each strategy and to better disentangle the effects.

Next, we attempt to disentangle the effects of the three strategies described in Section 3. Table 3 presents the average performance improvements when each strategy is applied separately as well as if two or more strategies are considered together. Improvements are measured based on the WLS_S hierarchical combinations when a single method (ETS or ARIMA) was used to produce the base forecasts. We observe that combining across methods (and not just aggregation levels) brings the largest improvements (around 2% on average), followed by the bias-adjustment strategy (on average 1.3% improvement). Performance improvements are similar for both forecast accuracy (MASE) and bias (SME). We observe minimal interactions between the strategies, with the average improvement of multiple strategies applied at the same time being roughly equal to the sum of the average improvements when each strategy was considered separately.

Table 3. Average performance improvements when each strategy is applied separately or in conjuction with other strategies.

Combination of methods	Bias adjustment	Avoiding seasonal shrinkage	MASE improvement	SME improvement
✓			1.98%	2.36%
	✓		1.29%	1.27%
		✓	0.54%	0.30%
✓	✓		3.22%	3.41%
✓		✓	2.18%	2.67%
	✓	✓	1.78%	1.54%
✓	✓	✓	3.89%	3.95%

9) For how many time series yields the third strategy (seasonal shrinkage) different results?

In Section 4 we note that the third strategy is applied to 150 out of the 2045 series in the sample. We also note that, although the improvements of this strategy have been identified as not statistically different, this could be due to the limited sample of series it is applied to. Thus, we conclude that more evidence is needed to evaluate its true potential.

Other remarks:

- page 3: figure -> Figure Fixed.
- page 9+13: section -> Section Fixed.
- page 9: descreibed -> described Fixed, thanks!
- page 9+12+13: sub-section 3.x -> Section 3.x Fixed.
- page 10+12+13: Fig 3/5/6 -> Figure 3/5/6 Now fixed.

- page 10: two strategies -> three strategies ? Fixed.
- The labels on Figures 3-6 are too small. We have now increase the size of the labels.
- Ref 10: doi looks broken Fixed, thanks!
- Ref 19+20: ";." at the end? Both are now fixed.
