# Supplementary material for: Improving the forecasting performance of temporal hierarchies
Source: PLoS One. 2019 Oct 3;14(10):e0223422. doi: 10.1371/journal.pone.0223422 (PMC6776316; doi:10.1371/journal.pone.0223422)
Supplement: Peer review history

# Peer review: Decision Letter 0

**DOI:** 10.1371/journal.pone.0223422.r001

**Authors:** Antonio Calcagnì

2 Sep 2019

[EXSCINDED]

PONE-D-19-19038

Improving the forecasting performance of temporal hierarchies

PLOS ONE

Dear Dr. Petropoulos,

Thank you for submitting your manuscript to PLOS ONE. After careful consideration, we feel that it has merit but does not fully meet PLOS ONE’s publication criteria as it currently stands. Therefore, we invite you to submit a revised version of the manuscript that addresses the points raised during the review process.

I have appreciated that your manuscript addresses the problem of how temporal hierarchical forecasting can be improved but there were some concerns which need to be addressed before final acceptance. Please see the attached reviewer comments for further details about necessary revisions. When revising your manuscript, please consider all issues mentioned in the reviewers' comments carefully.

We would appreciate receiving your revised manuscript by Oct 04 2019 11:59PM. When you are ready to submit your revision, log on to https://www.editorialmanager.com/pone/ and select the 'Submissions Needing Revision' folder to locate your manuscript file.

If you would like to make changes to your financial disclosure, please include your updated statement in your cover letter.

To enhance the reproducibility of your results, we recommend that if applicable you deposit your laboratory protocols in protocols.io, where a protocol can be assigned its own identifier (DOI) such that it can be cited independently in the future. For instructions see: http://journals.plos.org/plosone/s/submission-guidelines#loc-laboratory-protocols

Please include the following items when submitting your revised manuscript:

- A rebuttal letter that responds to each point raised by the academic editor and reviewer(s). This letter should be uploaded as separate file and labeled 'Response to Reviewers'.
- A marked-up copy of your manuscript that highlights changes made to the original version. This file should be uploaded as separate file and labeled 'Revised Manuscript with Track Changes'.
- An unmarked version of your revised paper without tracked changes. This file should be uploaded as separate file and labeled 'Manuscript'.

Please note while forming your response, if your article is accepted, you may have the opportunity to make the peer review history publicly available. The record will include editor decision letters (with reviews) and your responses to reviewer comments. If eligible, we will contact you to opt in or out.

We look forward to receiving your revised manuscript.

Kind regards,

Antonio Calcagnì, Ph.D.

Academic Editor

PLOS ONE

Journal Requirements:

1. When submitting your revision, we need you to address these additional requirements.

Please ensure that your manuscript meets PLOS ONE's style requirements, including those for file naming. The PLOS ONE style templates can be found at

http://www.journals.plos.org/plosone/s/file?id=wjVg/PLOSOne_formatting_sample_main_body.pdf and http://www.journals.plos.org/plosone/s/file?id=ba62/PLOSOne_formatting_sample_title_authors_affiliations.pdf

Reviewers' comments:

Reviewer's Responses to Questions

**Comments to the Author**

1. Is the manuscript technically sound, and do the data support the conclusions?

The manuscript must describe a technically sound piece of scientific research with data that supports the conclusions. Experiments must have been conducted rigorously, with appropriate controls, replication, and sample sizes. The conclusions must be drawn appropriately based on the data presented. 

Reviewer #1: Yes

**********

2. Has the statistical analysis been performed appropriately and rigorously? 

Reviewer #1: Yes

**********

3. Have the authors made all data underlying the findings in their manuscript fully available?

The PLOS Data policy requires authors to make all data underlying the findings described in their manuscript fully available without restriction, with rare exception (please refer to the Data Availability Statement in the manuscript PDF file). The data should be provided as part of the manuscript or its supporting information, or deposited to a public repository. For example, in addition to summary statistics, the data points behind means, medians and variance measures should be available. If there are restrictions on publicly sharing data—e.g. participant privacy or use of data from a third party—those must be specified.

Reviewer #1: Yes

**********

4. Is the manuscript presented in an intelligible fashion and written in standard English?

PLOS ONE does not copyedit accepted manuscripts, so the language in submitted articles must be clear, correct, and unambiguous. Any typographical or grammatical errors should be corrected at revision, so please note any specific errors here.

Reviewer #1: Yes

**********

5. Review Comments to the Author

Please use the space provided to explain your answers to the questions above. You may also include additional comments for the author, including concerns about dual publication, research ethics, or publication ethics. (Please upload your review as an attachment if it exceeds 20,000 characters)

Reviewer #1: The authors propose and discuss strategies to improve the performance of temporal hierarchical forecasting. They motivate their work by outlining drawbacks of current approaches and subsequently propose strategies to address them. The evaluation of the proposed methods on standard datasets underlines the effectiveness of the strategies.

I enjoyed reading the papers as it addresses an interesting and important topic and is generally well written. Overall, the paper is suitable for publication in PLOS ONE, but I have a few suggestions that would improve the paper.

1) Computational costs are mentioned in Section 3.1. How is this a (practically) relevant criteria for the rather short and monthly time series? How do the different strategies affect the total runtime? How much runtime do they add to the initial forecasts?

2) Where does Equation 5 come from? A more detailed explanation could be beneficial.

3) Why is the third strategy called seasonal shrinkage (damping seasonality)? Is it not just an indicator when temporal hierarchical forecasting, which would shrink the seasonality, should not be applied?

4) How does the temporal hierarchy which is considered for empirical evaluation look like? The fact that the forecast horizon covers 18 months (more than one year) and that the last 2 (4) predictions are excluded (in some cases) makes different hierarchies possible.

5) Why is the mean not used for bias-adjustments as introduced in Equations 3+4? You can still mention that the results are similar using the median operator.

6) Why are the results not tested for statistical significance?

7) Are the results comparable for SMAPE, which is also a frequently used accuracy measure?

8) While the result section contains all relevant information and the content is also structured, the structure can be improved. I would recommend making a better effort to disentangle the effects of the different strategies. This would also help to emphasize the answer to the initial research question: "What affects the performance?" All strategies improve the results, but how do they interact? Which is the most important strategy?

9) For how many time series yields the third strategy (seasonal shrinkage) different results?

Other remarks:

- page 3: figure -> Figure

- page 9+13: section -> Section

- page 9: descreibed -> described

- page 9+12+13: sub-section 3.x -> Section 3.x

- page 10+12+13: Fig 3/5/6 -> Figure 3/5/6

- page 10: two strategies -> three strategies ?

- The labels on Figures 3-6 are too small.

- Ref 10: doi looks broken

- Ref 19+20: ";." at the end?

**********

6. PLOS authors have the option to publish the peer review history of their article (what does this mean?). If published, this will include your full peer review and any attached files.

If you choose “no”, your identity will remain anonymous but your review may still be made public.

**Do you want your identity to be public for this peer review?** For information about this choice, including consent withdrawal, please see our Privacy Policy.

Reviewer #1: No

[NOTE: If reviewer comments were submitted as an attachment file, they will be attached to this email and accessible via the submission site. Please log into your account, locate the manuscript record, and check for the action link "View Attachments". If this link does not appear, there are no attachment files to be viewed.]

While revising your submission, please upload your figure files to the Preflight Analysis and Conversion Engine (PACE) digital diagnostic tool, https://pacev2.apexcovantage.com/. PACE helps ensure that figures meet PLOS requirements. To use PACE, you must first register as a user. Registration is free. Then, login and navigate to the UPLOAD tab, where you will find detailed instructions on how to use the tool. If you encounter any issues or have any questions when using PACE, please email us at figures@plos.org. Please note that Supporting Information files do not need this step.

# Author comment: Author response to Decision Letter 0

**DOI:** 10.1371/journal.pone.0223422.r002

9 Sep 2019

Please, see the respective attached document.

Attachment Submitted filename: PONE-D-19-19038 responses to comments.pdf Click here for additional data file.

# Editor report: Decision Letter 1

**DOI:** 10.1371/journal.pone.0223422.r003

**Authors:** Antonio Calcagnì

23 Sep 2019

Improving the forecasting performance of temporal hierarchies

PONE-D-19-19038R1

Dear Dr. Petropoulos,

We are pleased to inform you that your manuscript has been judged scientifically suitable for publication and will be formally accepted for publication once it complies with all outstanding technical requirements.

Within one week, you will receive an e-mail containing information on the amendments required prior to publication. When all required modifications have been addressed, you will receive a formal acceptance letter and your manuscript will proceed to our production department and be scheduled for publication.

Shortly after the formal acceptance letter is sent, an invoice for payment will follow. To ensure an efficient production and billing process, please log into Editorial Manager at https://www.editorialmanager.com/pone/, click the "Update My Information" link at the top of the page, and update your user information. If you have any billing related questions, please contact our Author Billing department directly at authorbilling@plos.org.

If your institution or institutions have a press office, please notify them about your upcoming paper to enable them to help maximize its impact. If they will be preparing press materials for this manuscript, you must inform our press team as soon as possible and no later than 48 hours after receiving the formal acceptance. Your manuscript will remain under strict press embargo until 2 pm Eastern Time on the date of publication. For more information, please contact onepress@plos.org.

With kind regards,

Antonio Calcagnì, Ph.D.

Academic Editor

PLOS ONE

# Editor report: Acceptance letter

**DOI:** 10.1371/journal.pone.0223422.r004

**Authors:** Antonio Calcagnì

25 Sep 2019

PONE-D-19-19038R1 

Improving the forecasting performance of temporal hierarchies 

Dear Dr. Petropoulos:

I am pleased to inform you that your manuscript has been deemed suitable for publication in PLOS ONE. Congratulations! Your manuscript is now with our production department. 

If your institution or institutions have a press office, please notify them about your upcoming paper at this point, to enable them to help maximize its impact. If they will be preparing press materials for this manuscript, please inform our press team within the next 48 hours. Your manuscript will remain under strict press embargo until 2 pm Eastern Time on the date of publication. For more information please contact onepress@plos.org.

For any other questions or concerns, please email plosone@plos.org. 

Thank you for submitting your work to PLOS ONE.

With kind regards,

PLOS ONE Editorial Office Staff

on behalf of

Dr. Antonio Calcagnì 

Academic Editor

PLOS ONE
